# Hepatitis B virus suppresses complement C9 synthesis by limiting the availability of transcription factor USF-1 and inhibits formation of membrane attack complex: implications in disease pathogenesis

**DOI:** 10.1186/s12929-022-00876-1

**Published:** 2022-11-14

**Authors:** Ayana Baidya, Mousumi Khatun, Rajiv Kumar Mondal, Suchandrima Ghosh, Bidhan Chandra Chakraborty, Shreya Mallik, S. K. Mahiuddin Ahammed, Abhijit Chowdhury, Soma Banerjee, Simanti Datta

**Affiliations:** 1 Centre for Liver Research, School of Digestive and Liver Diseases, Institute of Post Graduate Medical Education and Research (I.P.G.M.E.&R.), 244, A.J. C. Bose Road, Kolkata, 700020 India; 2 Multidisciplinary Research Unit, Institute of Post Graduate Medical Education and Research (I.P.G.M.E.&R.), 244, A. J. C. Bose Road, Kolkata, 700020 India; 3 Department of Hepatology, School of Digestive and Liver Diseases, Institute of Post Graduate Medical Education and Research (I.P.G.M.E.&R.), 244, A. J. C. Bose Road, Kolkata, 700020 India

**Keywords:** Complement C9, HBX, USF-1, Promoter hypermethylation, Membrane attack complex

## Abstract

**Background:**

The complement system functions primarily as a first-line host defense against invading microbes, including viruses. However, the interaction of Hepatitis B virus (HBV) with the complement-components during chronic HBV infection remains largely unknown. We investigated the mechanism by which HBV inhibits the formation of cytolytic complement membrane-attack complex (MAC) and studied its impact on MAC-mediated microbicidal activity and disease pathogenesis.

**Methods:**

Blood/liver tissues were collected from chronically HBV-infected patients and controls. HepG2^hNTCP^ cells were infected with HBV particles and Huh7 cells were transfected with full-length linear HBV-monomer or plasmids containing different HBV-ORFs and expression of complement components or other host genes were evaluated. Additionally, ELISA, Real-time PCR, Western blot, bioinformatics analysis, gene overexpression/knock-down, mutagenesis, chromatin immunoprecipitation, epigenetic studies, immunofluorescence, and quantification of serum HBV-DNA, bacterial-DNA and endotoxin were performed.

**Results:**

Among the MAC components (C5b-C9), significant reduction was noted in the expression of C9, the major constituent of MAC, in HBV-infected HepG2^hNTCP^ cells and in Huh7 cells transfected with full-length HBV as well as HBX. C9 level was also marked low in sera/liver of chronic hepatitis B (CHB) and Immune-tolerant (IT) patients than inactive carriers and healthy controls. HBX strongly repressed C9-promoter activity in Huh7 cells but CpG-island was not detected in C9-promoter. We identified USF-1 as the key transcription factor that drives C9 expression and demonstrated that HBX-induced hypermethylation of USF-1-promoter is the leading cause of USF-1 downregulation that in turn diminished C9 transcription. Reduced MAC formation and impaired lysis of HBV-transfected Huh7 and bacterial cells were observed following incubation of these cells with C9-deficient CHB sera but was reversed upon C9 supplementation. Significant inverse correlation was noted between C9 concentration and HBV-DNA, bacterial-DNA and endotoxin content in HBV-infected patients. One-year Tenofovir therapy resulted in improvement in C9 level and decline in viral/bacterial/endotoxin load in CHB patients.

**Conclusion:**

Collectively, HBX suppressed C9 transcription by restricting the availability of USF-1 through hypermethylation of USF-1-promoter and consequently hinder the formation and lytic functions of MAC. Early therapy is needed for both CHB and IT to normalize the aberrant complement profile and contain viral and bacterial infection and limit disease progression.

**Supplementary Information:**

The online version contains supplementary material available at 10.1186/s12929-022-00876-1.

## Background

Hepatitis B virus (HBV) is a small enveloped DNA virus that specifically infects the human hepatocytes and its persistent infection can cause various degrees of liver inflammation and damage that may eventually culminate in cirrhosis and even hepatocellular carcinoma (HCC) [1]. HBV genome is a partially double-stranded circular DNA of about 3.2 kilobase (kb) pairs, characterized by four overlapping open reading frames (ORFs), namely (i) ORF-P encoding HBV polymerase (P); (ii) pre-S1/pre-S2/S ORF encoding three envelope proteins, large/middle/small (HBsAg) surface antigen, (iii) pre-C/C–ORF coding for hepatitis B e antigen (HBeAg) and core protein (HBc) and (iv) X-ORF coding for the HBX protein, each having defined role in HBV life-cycle and disease pathogenesis. It is widely accepted that the outcome of HBV infection is largely determined by immune-mediated host–virus interactions [2]. The host complement system represents a crucial component of innate immunity and is a first-line defender against varied invading microorganisms, including the enveloped viruses. It comprises of a complex network of plasma and membrane-associated proteins, which are primarily produced by the hepatocytes and their activation leads to robust and efficient proteolytic cascades, which result in elimination of the pathogen and infected cells, generation of inflammatory response as well as modulation of the adaptive immunity [3]. Three biochemical pathways activate the complement system- the classical-, alternative- and lectin-pathway and all share a common terminal pathway that culminates in the formation of a multi-protein lytic complex called the membrane-attack complex (MAC) [4]. MAC assembly commences with the generation of C5 convertase, a protease that cleaves the complement-component C5 into two fragments, C5a and C5b. Complement C6 captures a labile binding site in C5b, followed by C7 association that renders the C5b-7 complex lipophilic and it in turn, attaches to and inserts into the target membrane. C5b-7 then binds the heterotrimeric C8αβγ to form C5b-8 complex, which then binds sequentially to 12–18 copies of C9 [3]. Together, these form the final mature C5b-9 MAC complex that is a hydrophilic protein channel, which disrupts the membrane integrity, leads to osmotic imbalance and ultimately causes lysis of pathogen and infected cells [5]. A number of complement regulatory proteins anchored on the host cell surface function to protect host tissues from bystander injury when complement is activated [3]. The invading microbes, on the other hand, have evolved mechanisms for evasion or dysregulation of the complement system to enhance their infectivity and even exacerbate the disease symptoms. These include cleaving complement proteins by encoding protease, inhibiting the synthesis of complement proteins, encoding complement regulatory proteins or incorporation of host complement regulators into virions [6]. In case of HIV-1, the presence of complement regulator CD59 in the viral envelope prevents complement-mediated lysis of the virus [7] while HCV core had been shown to transcriptionally downregulate complement expression in hepatocytes [8]. However, the interaction of HBV with the complement-components remains largely unexplored. Available studies indicated that serum levels of C3 and C4 were significantly decreased in patients with chronic HBV infection (CHI) [9]. Further, higher incidence of bacterial infection was observed among treatment-naïve chronically HBV-infected patients [10], suggesting that CHI may be linked with functional complement defects. MAC is the principal innate immune effector of the complement terminal pathway and the present study provides novel insights into the mechanism by which HBV attenuates the expression of C9, the major constituent of MAC, and thereby suppresses the formation and microbial activity of MAC, leading to viral persistence as well as increased bacterial infection in the setting of CHI. Understanding these processes will help in formulating appropriate therapeutic strategies to improve complement function and prevent disease progression in chronically HBV-infected patients.

## Methods

### Cloning and sequencing of full-length HBV and different HBV-ORFs

From an archival serum sample of a treatment-naïve CHB patient infected with HBV of genotype D, HBV-DNA was extracted using QIAamp DNA Mini kit (Qiagen, CA, USA). Full-length HBV genome (~ 3.2 kb) was amplified from the extracted DNA by high-fidelity Taq DNA polymerase (Thermo Fisher Scientific, MA, USA) and primers HBVP1 and HBVP2 (Additional file 1: Table S1), each bearing unique SapI restriction enzyme site [11]. The PCR product was purified by QIAquick gel extraction kit (Qiagen) and cloned into pJET1.2/blunt vector with CloneJET PCR cloning kit (Thermo Fisher Scientifc). Complete nucleotide sequences of three HBV clones were determined using BigDye terminator v3.1 cycle sequencing kit (Applied Biosystems) and different internal primers (Additional file 1: Table S1) on an automated DNA sequencer (3130, Genetic analyzer, Applied Biosystems). The sequences were compared with representative sequences of HBV D-genotype retrieved from GenBank to reconfirm their genotypic affiliations and one of the HBV/D clones was used for subsequent experiments. The sequence of that HBV/D clone is available in GenBank under Accession Number MF618339. Additionally, the complete PreS1/PreS2/S-ORF and ORF-P were PCR amplified from HBV/D clone with specific primer pairs (Additional file 1: Table S1) and cloned into pEGFP-N1 and pcDNA3.1/myc-His(B) vectors at HindIII/PstI and KpnI/HindIII restriction enzyme sites respectively (Additional file 1: Table S1) to generate pEGFPN1-HBs and pcDNA3.1/myc-His(B)-HBV-P clones. The clones were verified by sequencing. Plasmids pcDNA3.1/myc-His(B)-HBx and pCMV-HBc comprising HBX and core genes respectively [12] were gifted by Prof. Soma Banerjee.

### Cell culture and transfection

Human hepatoma cells Huh7 were cultured in Dulbecco’s modified Eagle’s medium (DMEM) (Hi-Media Laboratories Pvt. Ltd.) containing 10% fetal bovine serum (FBS) (Thermo Fisher Scientific). The full-length HBV linear monomer was released from pJET1.2/blunt vector by digestion with SapI enzyme (Thermo Scientific) at 37 °C for 12 h followed by gel purification using QIAquick gel extraction kit (Qiagen). The released HBV monomer or plasmid containing HBV-ORF was transfected individually into Huh7 cells seeded in 12 well plate at a concentration of 2 × 10^5^ cells/well using Lipofectamine 3000 (Invitrogen). Untransfected or empty vector transfected Huh7 cells were maintained as control. Six hours post transfection, the media was replaced with fresh DMEM media containing 10% FBS and after seventy-two hours transfected as well as untransfected Huh7 cells were harvested. All experiments were performed in triplicate and repeated at least three times. The Huh7 cell line was authenticated by STR profile analysis method from National Centre for Cell Science, Pune, India.

### HBV production and infection

HepG2.2.15 and HepG2^hNTCP^ cell lines were kindly gifted by Prof. Shyam Kottilil, Institute of Human Virology, University of Maryland. HepG2.2.15 cells (stably transfected with the complete HBV genome) were cultured in DMEM containing 10% FBS for 4 days. The supernatant containing HBV particles was collected and HBV stock titre (genome equivalents per millilitre) was assessed by Real Time PCR. HepG2^hNTCP^ cells expressing human sodium taurocholate cotransporting polypeptide (NTCP) are susceptible to HBV infection and HBV derived from culture supernatant of HepG2.2.15 cells was used as an inoculum for infection of HepG2^hNTCP^ cells. The HepG2^hNTCP^ cells were seeded in DMEM supplemented with 10% FBS and 1% L-Glutamine and after 72 h, the cells were infected with HBV particles (at 100 virus genome equivalent per cell) combined with 8% polyethylene glycol (PEG) 8000 and 2.5% dimethyl sulfoxide (DMSO) [13]. Six hours after infection the cells were washed with PBS, cultured in DMEM with 10% FBS, 1% L-Glutamine and 2.5% DMSO for 72 h and then harvested for further experiments. HBV uninfected HepG2^hNTCP^ cells served as control.

### Real-time PCR

Total RNA was extracted from harvested Huh7 and HepG2^hNTCP^ cells using TRIzol (Invitrogen) and cDNA was generated by reverse transcription with RevertAid Reverse Transcriptase (RT) enzyme (Thermo Scientific). The mRNA expression of different complement components (C5-C9) as well as that of other host genes were determined by Real-time PCR using the cDNA, SYBR Green Master mix (Applied Biosystems), and primer-pairs specific to each gene (Additional file 1: Table S2). Each sample was assessed in triplicates and the values were normalized by 18S rRNA expression level.

### Study subjects and samples

Treatment-naïve chronically HBV infected patients (HBsAg positivity > 6 months) representing different phases of chronic HBV infection were recruited from Hepatology Clinic of School of Digestive and Liver Diseases (SDLD), Institute of Post Graduate Medical Education and Research (I.P.G.M.E.&R.), Kolkata, India and were categorized into distinct cohorts namely-

#### Immune tolerant (IT)

Patients having HBeAg-positivity, high HBV-DNA (> 10^6^ IU/ml), normal serum alanine transaminase (ALT) levels (≤ 40 IU/L) and minimal or no necro-inflammation.

#### HBeAg-positive chronic hepatitis B (CHB)

Patients with high HBV-DNA > 10^4^ copies/ml, HBeAg-positive, elevated ALT > 40 IU/L and evidence of active hepatic necro-inflammation.

#### Inactive carrier (IC)

Patients negative for HBeAg and having low HBV-DNA (< 10^4^ copies/ml), normal ALT (≤ 40 IU/L) and minimal or no necro-inflammation.

#### HBeAg-negative chronic hepatitis B

Patients having HBV-DNA > 10^4^ copies/ml, HBeAg-negative, ALT > 40 IU/L and evidence of active hepatic necro-inflammation.

Patients with HIV/HCV co-infection, significant co-morbidity like diabetes mellitus, chronic alcoholism, intravenous drug abuse or any prolonged (> 7 days) drug therapy, evidences of any carcinoma, overt infection or autoimmune disorders were excluded. As comparison/control group sera samples were collected from healthy volunteers (HC) without any viral/bacterial infection and chronic/acute illness in preceding 6 months.

Five millilitre blood was collected from each study subject and sera was isolated, aliquoted and stored at − 80 °C until use. Prior informed written consents were obtained from the participants or from parents or legal guardians of minor participants prior to study inclusion. The access to human samples and all experimental protocols were carried out in accordance with the approved guidelines of the Ethical Review Committee of I.P.G.M.E.&R.

Liver biopsy tissue samples were collected from selected CHB patients during Endoscopic ultrasound-guided routine biopsy as a part of their diagnostic workup. In addition, liver tissues collected from patients undergoing cholecystectomy and those depicting normal liver histology and absence of liver metastasis served as control group. The tissues were collected in RNAlater solution (Invitrogen), kept at 4 °C overnight and then preserved at − 80 °C for future use.

### Measurement of C9 level in patient sera

Sera from chronically HBV infected patients and HC were used for estimation of C9 level by commercially available ELISA kits (Elabscience).

### Expression of C9 in liver tissue

The liver tissues stored in RNA later were homogenized, RNA was extracted using TRIzol reagent, cDNA was generated and the expression of C9 mRNA was determined by Real-time PCR as described previously.

### Western blot analysis

Huh7 cells transfected with pcDNA3.1/myc-His(B)-HBx or empty-vector were lysed in RIPA buffer [150 mM NaCl, 1% NP40, 0.5% Sodium deoxycholate, 0.1% SDS, 50 mM Tris (pH 8.0)] supplemented with 1X protease inhibitor cocktail (Roche, Switzerland). Protein concentrations in the whole cell lysates were measured by Bradford protein assay using Bradford reagent (Sigma Aldrich, MO, USA). Equal amount of total proteins were resolved on sodium dodecyl sulfate polyacrylamide gel electrophoresis (SDS PAGE), transferred to nitrocellulose membranes which was blocked using 5% BSA at room temperature for 1 h, and then treated with mouse anti-human anti-C9 (1:500) or mouse anti-human anti-USF-1 (1:500) primary antibody for overnight at 4 °C (Santa Cruz Biotechnology). The membranes were washed by PBST [PBS plus 0.1% Tween-20] and incubated in horseradish peroxidase (HRP)-labelled goat anti-mouse IgG (1:1000) at room temperature for 1 h, and then treated with chemiluminescent HRP substrate (Thermo Fisher Scientific). Finally, X-ray films were exposed, developed, dried, scanned and analysed with ImageJ software. Cellular α-tubulin served as loading control for the Western blot*.*

### Treatment of HBx transfected Huh7 cells with 5-Aza-2′-deoxycytidine and Trichostatin A

To determine the role of DNA methylation and histone deacetylation in C9 downregulation, HBx transfected Huh7 cells were treated with 5 μM DNA methylation inhibitor, 5-Aza-2′-deoxycytidine or 0.1 μM histone deacetylation inhibitor, Trichostatin A (TSA) while untreated cells served as control. At first, fresh DMEM media containing 5 μM 5-Aza-2′-deoxycytidine or 0.1 μM TSA were added separately to the Huh7 cells at the time of seeding the cells onto cell culture plates. Huh7 cells were then transfected with pcDNA3.1/myc-His(B)-HBx and 6 h after transfection, the cells were washed with PBS and the culture medium was replaced with media containing 5 μM 5-Aza-2′-deoxycytidine or 0.1 μM TSA. The media was replaced every 24 h with fresh media containing the same concentration of 5-Aza-2′-deoxycytidine or TSA. Seventy-two hours post transfection, the cells were harvested, total RNA was extracted and cDNA was prepared as before. The mRNA expression of C9 and USF-1 from both 5-Aza-2′-deoxycytidine and TSA treated cells were analyzed by Real-time PCR and compared with untreated cells.

### Mutagenesis of C9 promoter sequence

The C9 promoter region (− 786 to + 40 bp) [8] was first amplified from Huh7 genomic DNA with primers C9-prom_F and C9-prom_R (Additional file 1: Table S2) and inserted into KpnI/HindIII sites of pGL3-Basic vector (Promega, WI, USA) to generate the pGL3-C9-Prom construct. In the pGL3-C9-Prom construct, USF-1 binding site, E-box (CACGTG) was deleted to create pGL3-C9-Prom-mt plasmid with Agilent Site Directed Mutagenesis Kit (Agilent, CA, USA) and were confirmed by sequencing. The oligonucleotides used for the generation of mutated promoter construct are listed in Additional file 1: Table S2.

### Dual luciferase reporter assay

To study C9 promoter activity in presence of HBX, Huh7 cells were co-transfected with pGL3-C9-Prom, pcDNA3.1/myc-His(B)-HBx and pRL-CMV *Renilla Luciferase* Reporter vector (Promega, WI, USA). Seventy-two hours post transfection, the cells were harvested in Passive Lysis Buffer (Promega) and the *firefly* and *Renilla* luciferase activities were measured sequentially using the Dual-Luciferase Reporter Assay kit (DLR) (Promega) in a luminometer (Promega). The activity of *Renilla* luciferase was used to normalize the *firefly* luciferase enzyme activity.

### Analysis of CpG island distribution and transcription factors (TF) binding at C9 promoter

Putative CpG islands in the promoter region of C9 were analyzed using online tool Methprimer [14].

Transcription factors (TF) binding at the promoter region of C9 were identified bioinformatically with PROMO and TFBIND software using threshold of < 5% dissimilarity and string length > 10. The level of expression of TFs in liver was determined from Human Protein Atlas database. In vitro studies were performed with selected hepatic TFs binding to C9 promoter.

### Overexpression and knockdown of USF1

The pCMV-USF1 plasmid encoding USF-1 was a generous gift of Prof. Marie-Dominique Galibert. Huh7 cells were transfected with pcDNA3.1/myc-His(B)-HBx, pGL3-C9-Prom and pRL-CMV Renilla Luciferase Reporter vector along with different concentrations (0, 400, 800 and 1200 ng/ml) of pCMV-USF1 plasmid and luciferase activity of C9 promoter was assessed from the harvested cells. The mRNA expression of C9 was also measured from Huh7 cells co-transfected with pcDNA3.1/myc-His(B)-HBx and pCMV-USF1 plasmid (in different concentration). Additionally, USF-1 antisense oligonucleotide (USF-1_ASO) (Additional file 1: Table S2) directed against the exon1 of USF-1 was designed from USF-1 gene sequence obtained from UCSC genome browser. Huh7 cells were transfected with pGL3-C9-Prom, pRL-CMV Renilla Luciferase Reporter vector and USF-1_ASO (0–1200 ng/ml) and C9-promoter luciferase activity was measured as described earlier. Further, C9 mRNA expression was evaluated in Huh7 cells transfected with USF-1_ASO (1000 ng/ml) or scrambled oligo control (ASO_Ctrl) (1000 ng/ml) (Additional file 1: Table S2), having no known targets in HBV or human transcriptome.

### Chromatin immunoprecipitation (ChIP) assay

To clarify the interaction between USF-1 and the promoter region of C9, ChIP assay was performed using EpiQuik Chromatin Immunoprecipitation kit (EpigenTek, NY, USA) according to the manufacturer's instructions. Firstly, the assay strips were prepared by coating with mouse anti-human anti-USF1 antibody (Santa Cruz Biotechnology) and non-immune IgG (provided with the kit) that served as negative control. 2 × 10^6^ Huh7 cells were transfected with pcDNA3.1/myc-His(B)-HBx or empty vector and after 72 h the cells were collected and the chromatin was crosslinked with 1% formaldehyde. 1.25 M glycine was added for 5 min to terminate the crosslinking process. The cells were incubated in lysis buffer supplemented with 1X protease inhibitor cocktail (Roche). The cross-linked DNA–protein complexes were sheared into ~ 200–1000 bp fragment by sonication. 5% input reference was removed from the sonicated DNA–protein complexes. 100 μl of the supernatant from each sample were then added to the antibody coated assay strips for immunoprecipitation. The DNA crosslinks were reversed and the proteins were removed by treatment with Proteinase K at 65 °C for 2 h in all the sample tubes including the input DNA vials and DNA was recovered using DNA purification spin columns. Finally, the DNA was quantified by Real-time PCR using primers specific for the C9 promoter as listed in Additional file 1: Table S2. Data obtained were then normalized to the negative control and expressed as percent recovery relative to the input.

### Bisulfite sequencing

Genomic DNA was isolated from pcDNA3.1/myc-His(B)-HBx or empty vector transfected Huh7 cells using DNA extraction kit (FAVORGEN Biotech Corp., Taiwan). 2ug of isolated genomic DNA was bisulfite treated and purified using EZ DNA methylation kit (Zymo Research, CA, USA). The bisulfite-modified genomic DNA was amplified with bisulfite specific primers of USF-1 promoter (Additional file 1: Table S2) for 35 cycles of 95 °C for 30 s, 56 °C for 30 s, and 72 °C for 20 s and the amplified products were cloned into pJET1.2 blunt vector. Six different clones from each group pcDNA3.1/myc-His(B)-HBx /empty vector transfected cells were screened by sequencing and DNA methylation status was analyzed.

### Immunofluorescence

HBV transfected Huh7 cells were grown as monolayer on poly-l-lysine coated coverslips in 6-well plate for 48 h and then treated with 50 μl sera from HC or CHB patients or with CHB patient sera supplemented with recombinant C9 protein at final concentration of 15 ng/ml of culture media for 60 min at 37 °C. The cells were fixed with 4% paraformaldehyde for 10 min and after washing with ice cold TBS, the masked antigens were retrieved by treatment with 0.05% trypsin-calcium chloride solution [trypsin 0.5%, calcium chloride 1% (pH 7.8)] at 37 °C for 20 min. After washing with TBS containing 0.025% TritonX-100, blocking was done with 1% BSA in TBS for 2 h. The cells were stained overnight at 4 °C with 1:500 dilution of mouse anti human anti-C5b-9 primary antibody (Santa Cruz Biotechnology) followed by incubation with 1:1000 dilution of goat anti-mouse IgG secondary antibody conjugated to Alexa fluor 488 (Thermo Fisher Scientific) for 1 h at 37 °C. Pro-Long Gold Antifade reagent with 4′,6-diamidino-2-phenylindole (DAPI) (Cell Signaling Technology) was used for nuclear counterstaining and mounting. Images were captured in Thunder imager (Leica) at 40× magnification.

### Complement-mediated cytolysis assay

Huh7 cells were transfected with full-length HBV monomer and distributed in 96 well plate at a density of 1 × 10^4^ cells/well. After 3 days, the cells were treated with sera from HC or CHB patients or with CHB sera supplemented with recombinant C9 protein (final concentration 15 ng/ml) for 1 h at 37 °C. Finally, the cells were treated with 40 μg/well MTT solution (Sigma Aldrich) and incubated for 4 h at 37 °C in dark. Optical density of the solution was measured on an ELISA plate reader with a test wavelength of 570 nm and a reference wavelength of 630 nm.

### C9 deposition on *E. coli*

1 × 10^7^ cells of *E. coli* DH5α were incubated with CHB patient or HC sera at 37 °C for 60 min. After a wash with PBS, the bacteria were plated and adhered onto 96-well enzyme immunoassay (EIA) plates at ~ 5 × 10^6^ cells per well. The nonspecific binding sites were blocked with 200 µl of 0.5% bovine serum albumin (BSA)–PBS and incubated for 30 min at 37 °C. After washing the plates were sequentially incubated at room temperature with 100 µl of 1:100 (v/v) mouse anti-human anti-C9 antibody for 60 min, 100 µl of 1:1000 HRP-conjugated goat anti-mouse secondary antibody for 30 min followed by addition of 100 µl of substrate solution for visualizing HRP activity. The reaction was stopped with 50 µl of 10% H_2_SO_4_, and the colour reaction was detected by reading the optical density at 490 nm in ELISA reader.

### Bactericidal assay

1 × 10^4^ cells of *Escherichia coli* strain DH5α were incubated with 100 µl of sera (1:500 dilution) of CHB patient or HC or heat inactivated sera of HC (for complement inactivation) at 56 °C for 60 min. After washing, serial tenfold dilutions of bacterial suspensions were prepared in saline solution and 100 µl was spread on LB plates. Colony forming units (CFU) were counted from the plates after 16 h of incubation at 37 °C. The experiment was performed in triplicate and repeated thrice.

### Determination of bacterial load and endotoxin titre

Bacterial DNA in sera samples of CHB patients of different phases and healthy volunteers was isolated by incubation of the sera samples with lysozyme at 56 °C for 30 min followed by DNA isolation using QiaAmp Blood DNA mini kit (Qiagen). The V3–V4 hypervariable region of the bacterial 16S rRNA gene was amplified with primers 338F (5′ACTCCTACGGGAGGCAGCAG-3′) and 806R (5′-GGACTACHVGGGTWTCTAAT-3′) [15] using Real-time PCR. The bacterial load was determined from standard curve prepared using *E. coli* DNA of known bacterial load as standard. Endotoxin levels in sera of chronically HBV infected patients and healthy individuals were measured by ELISA method using Chromogenic Endotoxin Quant Kit (Thermo Fisher Scientific). Briefly, endotoxin standard solutions were prepared by reconstitution with endotoxin free water and then 50 μl of each standard as well as serum sample of HC and chronically HBV infected individuals were added to 96 well plate at 37 °C waterbath. Amoebocyte lysate reagent was added to each well and incubated at 37 °C, followed by addition of chromogenic substrate and finally reaction was stopped by 25% acetic acid. Optical density was measured at 405 nm in optical plate reader.

### Longitudinal assessment of serum C9 level and virological/bacteriological parameters in CHB patients receiving tenofovir

From 10 CHB patients treated with tenofovir (300 mg daily), blood samples were collected at day 0 before the initiation of therapy (= baseline) and at the end of 12 months of treatment respectively and serum C9, HBV DNA, ALT, bacterial load and endotoxin titre were assessed as described previously.

### Statistical analysis

Data were expressed as mean ± standard deviation (SD). Paired Student's *t* tests and Repeated Measures ANOVA were used to determine statistical significance. Comparison between groups was done by one‐way ANOVA followed by Tukey's Multiple Comparison Test. Linear regression was performed for correlation analysis. Statistical analysis was performed using GraphPad Prism Software, version 5.0. For all tests, *p* < 0.05 was considered statistically significant.

## Results

### Downregulation of C9 expression in HBV-transfected Huh7 cells, HBV-infected HepG2^hNTCP^ cells and in chronically HBV-infected patients

**Fig. 1:**
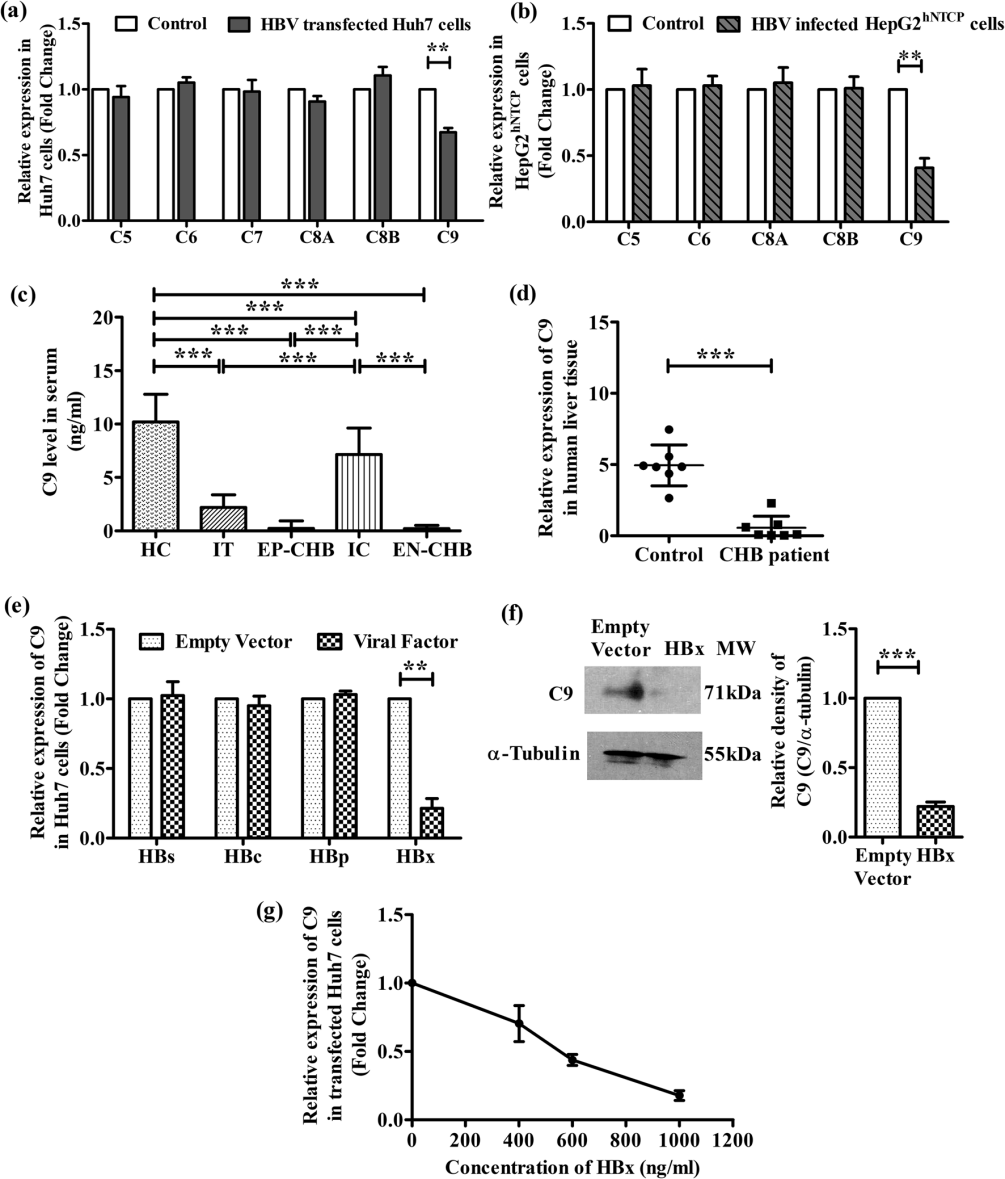
HBV diminishes complement C9 expression. Relative mRNA expression of different membrane attack complex components in **a** Huh7 cells transfected with full-length HBV linear monomer relative to untransfected cells (control) and **b** HepG2^hNTCP^ cells infected with cell culture-derived HBV particles in comparison to uninfected HepG2^hNTCP^ cells (control) as measured by Real-time PCR. **c** Serum concentration of C9 in IT, EP-CHB, IC, EN-CHB and HC detected by ELISA. **d** Relative mRNA expression of C9 in chronically HBV infected liver biopsy tissues compared to control liver tissues. **e** Relative mRNA expression of C9 in Huh7 cells transfected separately with plasmids encoding all three HBV surface proteins (HBs), HBV core (HBc), HBV Polymerase (HBp) or HBV X (HBx). **f** Expression of C9 protein in HBx or empty vector transfected Huh7 cells assayed by immunoblot with anti-C9 primary antibody; cellular α-Tubulin served as the loading control. **g** Relative mRNA expression of C9 when transfected with different concentrations (0, 400, 600 and 1000 ng/ml) of pcDNA3.1/myc-His(B)-HBx plasmid. mRNA expression was normalized with endogenous 18S ribosomal RNA value. In cases of **a**, **b** and **d**–**g**, values represent data from three independent experiments, mean ± SD. For **c** statistical significance was assessed by one-way ANOVA followed by Tukey's Multiple Comparison Test (**p < 0.005 and ***p < 0.0001). Paired *t*-tests were performed for comparisons of paired groups in a, b and d-f. **p < 0.005, ***p < 0.0001

To explore whether HBV could interfere with MAC formation, we first examined the expression of different MAC components (C5/C6/C7/C8/C9) in Huh7 cells transfected with full-length HBV by Real-time PCR. As indicated in Fig. 1a, a significant reduction in C9 expression (~ 1.5-fold) was perceived in HBV-transfected Huh7 cells as compared to untransfected cells while the expression of other components of MAC remained comparable. Further, we also investigated the expression of MAC components in HepG2^hNTCP^ cells infected with cell culture-derived HBV produced by stable HBV-replicating cells (HepG2.2.15). The results depicted a similar trend as seen in Huh7 cells, with a significant (~ 2.4 fold) downregulation in C9 expression in HBV-infected HepG2^hNTCP^ cells relative to uninfected control cells whereas no variation was observed in the expression of C5, C6 and C8 (Fig. 1b). C7 was undetectable in HepG2^hNTCP^ cells.

We next determined by ELISA, the level of C9 in the sera of HC and chronically HBV-infected patients in different disease phases, IT, EP-/EN-CHB and IC. The clinical and demographic features of study subjects are provided in Additional file 1: Table S3. C9 was found to be significantly low in IT, EP-CHB and EN-CHB relative to IC and HC (Fig. 1c). Further, when compared between IC and HC, C9 concentration was less in IC than HC. In addition, we detected that C9 transcript level in liver biopsy tissues of CHB patients was significantly diminished than control (Fig. 1d).

### HBX suppresses the expression of C9

To identify the viral factors responsible for the downregulation of C9, Huh7 cells were separately transfected with plasmids expressing different HBV proteins and the expression of C9 was evaluated by Real-time PCR. C9 mRNA level was found to be substantially low (~ 4.7 fold) in HBx-transfected Huh7 cells than in cells transfected with empty-vector (Fig. 1e). In contrast, cells expressing other HBV proteins showed no significant change in the amount of C9 mRNA relative to control. The down-regulatory effect of HBX on C9 expression was further confirmed at protein levels by immunoblot analysis of cell lysate with anti-C9 antibody and a marked reduction in C9 was noted as compared to control cells (Fig. 1f). To assess the specificity of inhibitory effect of HBX on C9 expression, Huh7 was transfected with increasing amounts of HBx-expressing plasmid and a corresponding dose-dependent diminution in C9 mRNA level was observed (Fig. 1g). Collectively, the results suggest that HBX resulted in a decline in C9 expression.

### HBV induced DNA hypermethylation contribute to C9 downregulation

**Fig. 2:**
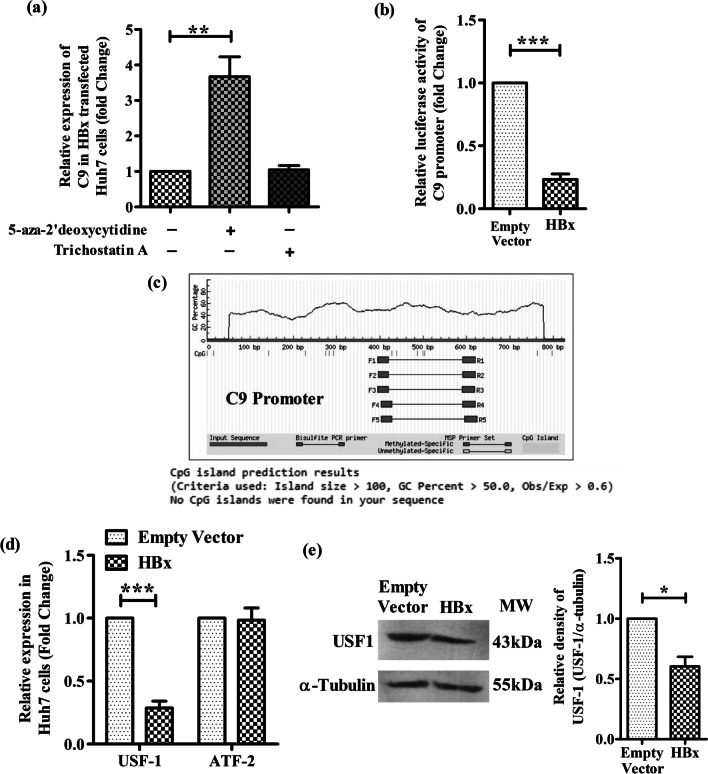
HBX downregulates C9 promoter activity and USF-1 expression. **a** Relative expression of C9 in HBx transfected Huh7 cells with or without DNA methylation inhibitor 5-aza-2’-deoxycytidine (5 μM) or histone deacetylation inhibitor Trichostatin A (0.1 μM). **b** Relative luciferase activity of C9 promoter-luciferase reporter construct (pGL3-C9-Prom) when co-transfected with HBx-expressing plasmid [pcDNA3.1/myc-His(B)-HBx] or empty vector. **c** CpG island prediction in C9 promoter using Methprimer online software. Relative expression of **d** USF1and ATF2 mRNA and **e** USF-1 protein in HBx- or empty vector transfected Huh7 cells measured respectively by real time PCR and western blot analysis using anti-USF1 antibody. Mean ± SD are presented based on three independent experiments. Paired t tests were performed for comparing paired groups. *p < 0.05, ***p < 0.0001

HBX induced epigenetic changes, including aberrations in DNA methylation patterns and histone modifications had been shown to play important roles in regulating the expression of a variety of host genes [16]. We tested whether C9 was silenced by HBX via perturbations in DNA methylation or histone deacetylation in Huh7 cells. For this, HBX-transfected Huh7 cells were treated with DNA methylation inhibitor, 5-Aza-2′-deoxycytidine or histone deacetylation inhibitor, trichostatin A (TSA) and C9 expression was analyzed. The expression of C9 was found to be significantly enhanced in presence of 5-Aza-2′-deoxycytidine as compared to the untreated cells while no effect was observed following TSA treatment, suggesting that C9 downregulation could be attributed to HBV mediated DNA hyermethylation (Fig. 2a).

### HBX inhibits C9 promoter activity

To further investigate the mechanism of HBV-mediated suppression of C9, we first examined the effect of HBX on the activity of the C9 promoter. A C9 promoter-luciferase reporter construct (pGL3-C9-Prom) was generated and transfected into Huh7 cells along with pcDNA3.1/myc-His-HBx or the empty-vector and the firefly luciferase activity was measured. In both cases, the co-transfected plasmid pRL-CMV, expressing *Renilla* luciferase provided an internal control and the luminescence of firefly luciferase was normalized to that of *Renilla* luciferase. The reporter assay revealed that the ectopic expression of HBX significantly decreased the luciferase signalling driven by C9 promoter by ~ 4.3 fold as compared to that of vector-control (Fig. 2b).

### Absence of CpG islands in C9 promoter

To examine the possibility of C9 promoter inactivation via HBX-induced hypermethylation, we first used MethPrimer program to locate CpG islands within the C9 promoter. However, in silico analysis revealed that CpG islands are absent in the promoter of C9 gene (Fig. 2c).

### HBX mediated alteration in the expression of TF binding to C9 promoter

Insufficient availability of TFs binding to a promoter is also considered to be an important cause for the downregulation of promoter activity and decrease in gene expression. Using online tools, PROMO and TFBIND and setting a cut-off of < 5% dissimilarity index and string length >  = 10, we first analyzed the TFs that bind to C9 promoter and identified four important TFs namely, USF-1, ATF2, FOXO4, STAT1β. However, out of these, only USF1 and ATF2 are expressed in hepatocytes as inferred from Human Protein Atlas data. So we studied the expression of ATF2 and USF-1 in HBx-transfected Huh7 cells by Real-time PCR. We noticed that HBX caused a significant reduction (~ 3.5 folds) in USF-1 mRNA in comparison to empty-vector transfected cells while no change was perceived in the expression of ATF2 (Fig. 2d). The inhibitory effect of HBX on USF-1 at protein level was also seen in Western blot analysis (Fig. 2e). This low USF-1 expression is likely associated with its decreased binding to the C9 promoter and the consequent attenuation in transcriptional activity.

### Enhancement in C9 promoter activity and C9 expression in HBx-transfected Huh7 cells upon USF-1 overexpression

**Fig. 3:**
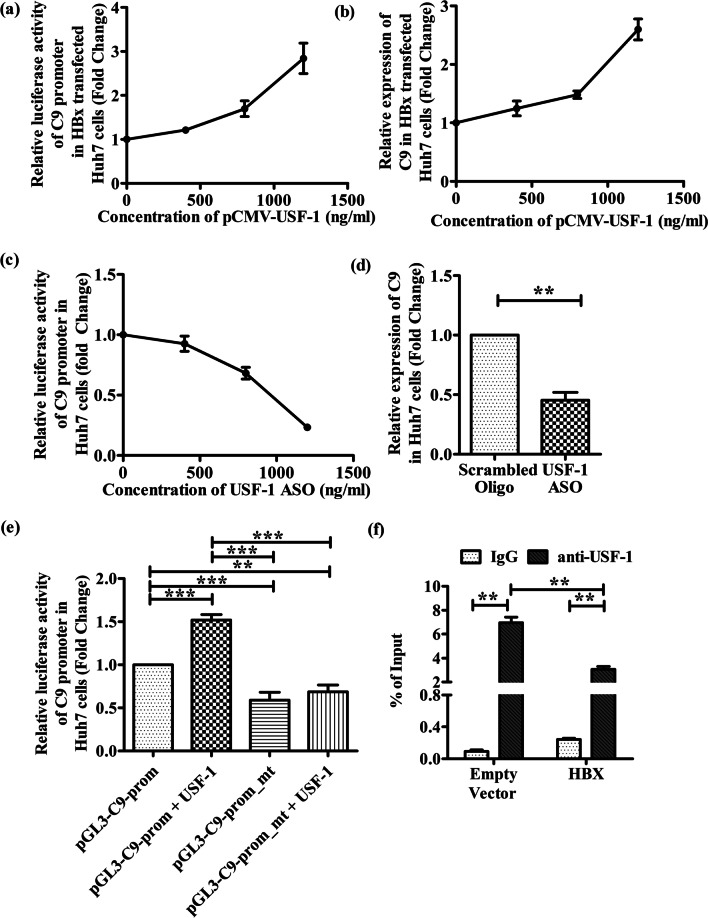
USF-1 is an important transcription factor that regulates C9 expression and is inhibited by HBX. **a** Relative luciferase activity of C9 promoter in Huh7 cells transfected with pcDNA3.1/myc-His(B)-HBx, pGL3-C9-Prom and pRL-CMV Renilla Luciferase Reporter vector along with different concentrations (0, 400, 800 and 1200 ng/ml) of pCMV-USF1 plasmid. **b** Relative C9 mRNA expression following transfection of Huh7 cells with pcDNA3.1/myc-His(B)-HBx and pCMV-USF1 plasmid in different concentrations (0, 400, 800 and 1200 ng/ml). **c** Relative C9 promoter luciferase activity in Huh 7 cells transfected with pGL3-C9-Prom, pRL-CMV Renilla Luciferase Reporter vector and different concentrations of antisense oligonucleotide designed against USF-1 gene (USF-1_ASO) (0–1200 ng/ml). **d** Relative C9 mRNA expression following transfection of Huh7 cells with USF-1_ASO (1000 ng/ml) or scrambled oligo control (ASO_Ctrl) (1000 ng/ml). **e** Assessment of relative C9 luciferase activity upon transfection of Huh7 cells with wild-type pGL3-C9-Prom and its mutated derivative pGL3-C9-Prom-mt in presence or absence of USF-1 expressing plasmid (pCMV-USF1). **f** ChIP analysis of USF1 binding to C9 promoter in HBx and empty vector transfected Huh7 cells. ChIP analysis was performed using IgG (negative control) and anti-USF-1 antibody. The relative enrichment of C9 promoter DNA was normalized to the input DNA (5%) for each experiment. Mean ± SD are based on three independent experiments. Paired t tests were performed for comparing paired groups in **d**, **f**. Comparisons between groups were performed using the one-way ANOVA with *p* values adjusted by the Tukey’s multiple comparison test in **e**. **p < 0.005, ***p < 0.0001

To determine whether USF-1 plays an important role in transcriptional regulation of C9, we reassessed the C9 promoter activity and C9 expression in HBx-transfected Huh7 cells in presence of different concentration of USF1-expressing pCMV-USF1 plasmid. As shown in Fig. 3a and b, increasing expression of USF-1 resulted in dose-dependent induction of C9 promoter as well as C9 mRNA in HBx-transfected cells. Moreover, we knocked down USF-1 in control Huh7 cells by an ASO and observed a progressive decrease in C9 promoter luciferase activity and C9 expression with increased concentration of ASO relative to scrambled oligo control (Fig. 3c, d). Together, these data suggest that USF-1 is a key regulatory factor for C9 gene expression in hepatocytes.

### USF-1 binds to the E-box elements of C9-promoter and the binding is reduced in presence of HBX

USF-1 is known to recognize and bind to the E-box motif that conforms to the core hexanucleotide consensus sequence of CANNTG within a gene promoter [17]. Sequence analysis of C9 promoter (nt − 786 to + 40) revealed the presence of a putative E-box site (5′-CACGTG-3′) located in nt. − 738/− 732. In order to examine the functional role of E-box motif in the basal transcriptional activity of the C9 promoter, the 6-bp consensus sequence was deleted by site-directed mutagenesis in pGL3-C9-Prom construct to generate pGL3-C9-Prom-mt. The wild-type promoter and the mutated derivative were transfected into Huh7 cells and assayed for luciferase activity. Results presented in Fig. 3e indicate that E-box deletion greatly lowered the basal activity of the C9 promoter. We moreover, studied the effect of overexpression of USF-1 on luciferase activities of both the promoter constructs. While pGL3-C9-Prom exhibited ~ 1.5-fold more robust promoter activity in response to increased cellular USF-1, no change was seen in the activity of pGL3-C9-Prom-mt even in presence of USF-1 (Fig. 3e). These results signify that USF-1 had an E-box-dependent stimulatory effect on C9 promoter. To directly assess the presence of USF-1 bound to C9 promoter, ChIP was performed in Huh7 cells transfected with pcDNA3.1/myc-His-HBx or the empty-vector. An enrichment of E-box containing C9 promoter DNA was detected when DNA was immunoprecipitated with anti-USF-1 antibody but not when IgG was used (Fig. 3f). However the occupancy of C9 promoter with USF-1 was found to be significantly reduced (~ 2.3 folds) in presence of HBX as opposed to vector-control (Fig. 3f). In all cases the immunoprecipitated DNA was quantified by Real-time PCR and the results were normalized to input DNA. Together these results illustrate that the binding of USF-1 to its cognate E-box element in C9 promoter contributes to the activation of C9 gene expression while HBX limits the availability of USF-1 for binding to the target promoter.

### HBX hypermethylates USF1 promoter and attenuates USF1 and C9 transcription

**Fig. 4:**
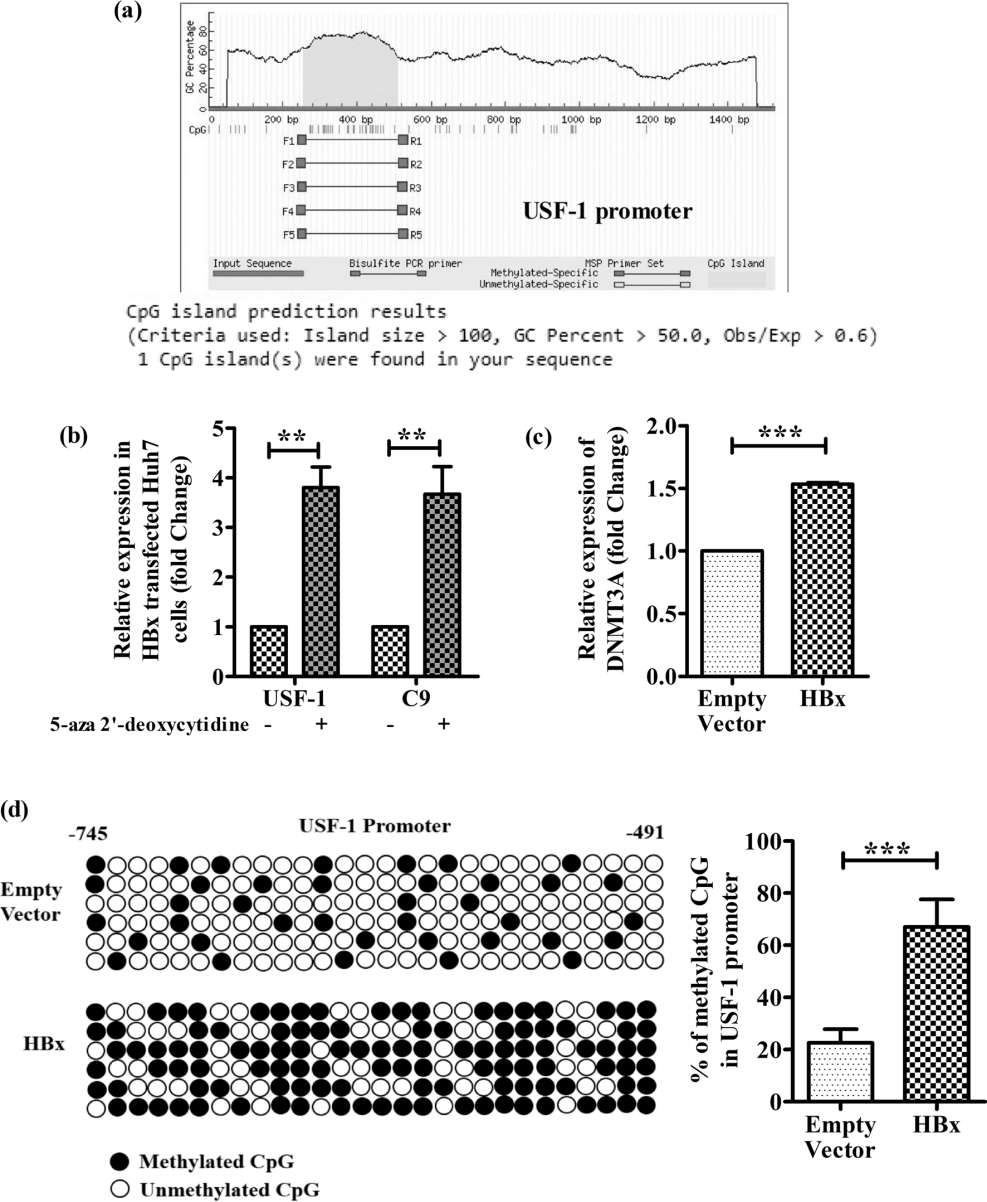
HBX limits USF-1 availability for C9 transcription by hypermethylation of USF-1 promoter. **a** CpG island prediction in USF-1 promoter using Methprimer online software. **b** Relative expression of USF1 and C9 in HBx transfected Huh7 cells with or without DNA methylation inhibitor 5-aza-2′-deoxycytidine (5 μM). **c** Relative expression of DNMT3A in Huh7 cells transfected with HBX-expressing plasmid and empty vector. **d** Schematic representation of CpG dinucleotides at USF-1 promoter in Huh7 cells transfected with HBX-expressing plasmid and empty vector. The horizontal line represents the sequencing result of one clone, and the vertical line represents each individual CpG sites. The open circles represent unmethylated cytosine and the closed circles represent methylated cytosine. The percentages of methylated CpGs in USF-1 promoter in case of HBx and empty vector transfected Huh7 cells were calculated and compared. Values represented as mean ± SD. Paired *t* tests were performed for comparing paired groups. **p < 0.005, ***p < 0.0001

The decreased binding of USF-1 to C9 promoter in HBx-transfected Huh7 cells could be attributed to the lower abundance of USF-1 in these cells and we sought to identify the molecular mechanism underlying the downregulation of USF-1 by HBX. Inspection of the promoter region (nt-1000 to + 40) of USF-1 gene using MethPrimer indicated the presence of CpG islands between nt -745 and − 491 (Fig. 4a) and we speculated that HBX-induced hypermethylation of USF-1 promoter may be responsible for the deficit of USF-1 gene expression. To confirm this hypothesis, HBX-expressing Huh7 cells were treated with 5-Aza-2′-deoxycytidine and a strong upregulation of USF-1 as well as C9 transcripts was observed relative to untreated cells (Fig. 4b). As expected, no change in USF-1 expression was found in TSA treated cells (Additional file 1: Fig. S1). We also examined in HBX-expressing Huh7 cells, the expression level of DNMT3A, which catalyzes the addition of methyl groups to cytosine residues of CpG dinucleotides. Our results indicated that DNMT3A expression was markedly enhanced upon HBX expression in Huh7 cells in comparison to vector-control (Fig. 4c). In addition, we evaluated by bisulfite sequencing, the DNA methylation patterns across USF-1 promoter in presence or absence of HBX. The frequency of USF-1 promoter methylation in HBx-expressing Huh7 cells was found to be significantly high (~ 67%) than that of control cells (~ 23%) (Fig. 4d). Together, these data suggest that HBX induces CpG methylation at USF-1 promoter that results in the downregulation of USF-1 and restricts its binding to the C9 promoter and thereby leads to suppression of C9 expression.

### Inhibition of MAC formation on HBV-transfected Huh7 cells and impaired cell lysis by sera from CHB patients

**Fig. 5:**
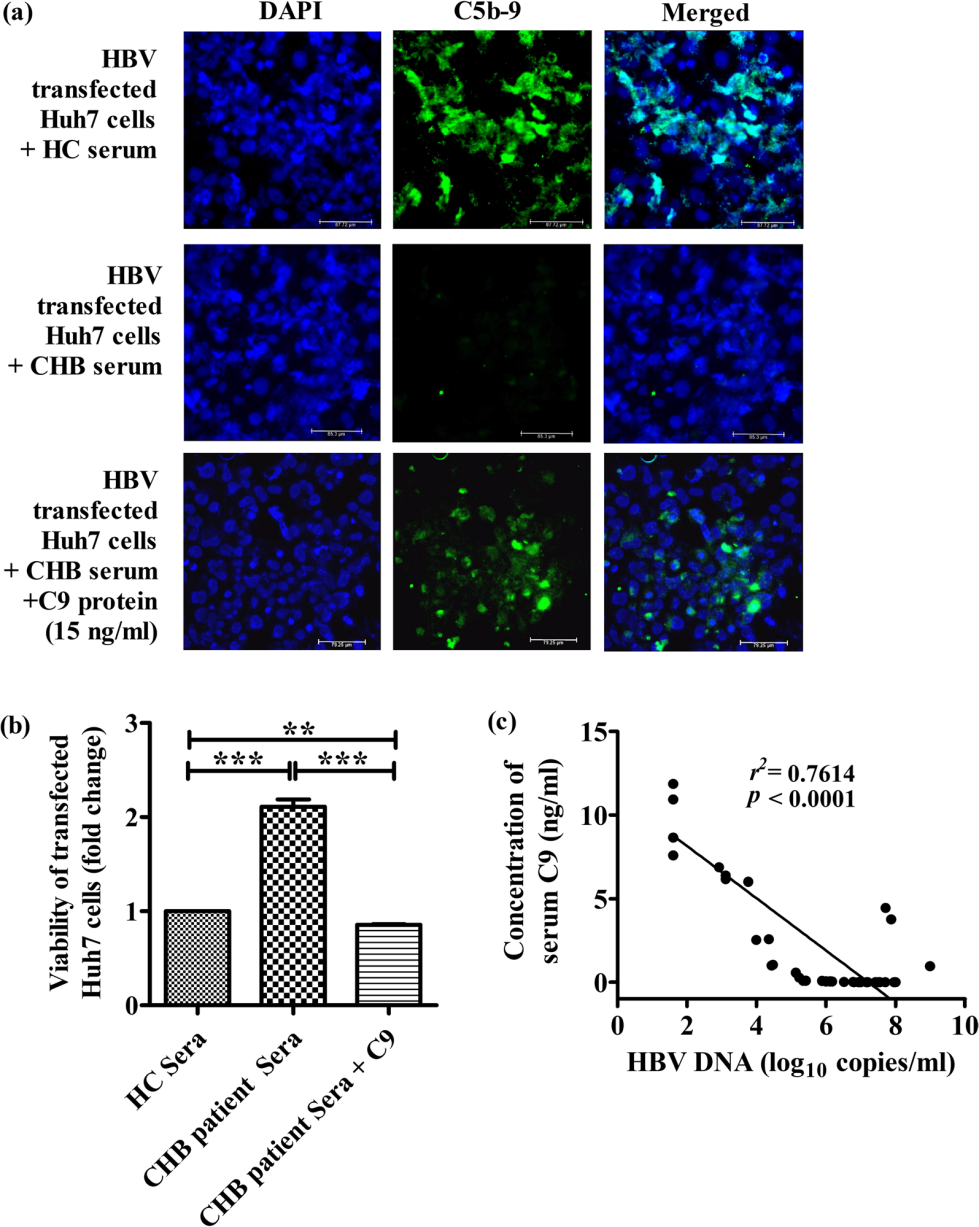
Reduced MAC formation and Huh7 cell lysis with CHB sera were reversed upon C9 supplementation. **a** Representative immunofluorescence images at ×40 magnification showing MAC formation (C5b-9, green) on HBV transfected Huh7 cells treated with serum from HC and CHB patient as well as CHB serum supplemented with C9 protein. The nucleus was counterstained with DAPI (blue). **b** Viability of HBV transfected Huh7 cells measured by MTT assay upon incubation with HC sera, CHB sera and C9 supplemented CHB sera. The absorbance readings were averages of triplicate experiments. **c** Correlation analysis between serum C9 concentration and HBV-DNA levels in chronically HBV infected individual. One-way ANOVA followed by Tukey’s multiple comparison were used in (b). **p < 0.005, ***p < 0.0001

We studied the impact of C9 deficiency on the assembly of MAC complex and its lytic function by incubating HBV-transfected Huh7 cells with sera from CHB patients and HC, which served as source of the complement-components. The localization of MAC on Huh7 cells was assessed by fluorescence imaging using anti-C5b-9 antibody. C5b-9 deposition was found to be extensive in HBV-transfected Huh7 cells treated with serum of HC while it was sparse or negligible in cells incubated with CHB patient serum that had diminished concentration of C9 (Fig. 5a). In agreement with the pattern seen in MAC formation, sera from CHB patients was also much less effective in causing cell lysis as evident from increased viability of the cells seen by MTT assay in comparison to HC sera that could induce pronounced lysis and consequently reduced viability of the transfected cells (Fig. 5b). Moreover, in vitro supplementation of a low dose of  15 ng/ml C9 protein in the serum of CHB patients resulted in substantial enhancement (~ 71%) in MAC accumulation on these cells (Fig. 5a) as well as considerable decline in cellular viability (~ 2.5 fold) (Fig. 5b), reinforcing the critical role of C9 in the generation and activity of MAC.

### Lower serum C9 concentration correlates with higher HBV-DNA in HBV-infected patients

We investigated the association between the serum C9 concentration and viral load in chronically HBV-infected patients in IT, EP-/EN-CHB and IC phases. A significant negative correlation was noted between serum C9 and HBV-DNA levels (*r*^*2*^ = 0.7614, *p* < 0.0001) (Fig. 5c), such that patients in IT/CHB, with low C9 showed high HBV titre, which was markedly reduced in IC, where C9 is relatively high. It is thus reasonable to assume that MAC formation on viral surface or on infected cells and their subsequent lysis play an important role in the reduction of HBV load.

### Impairment of bacterial killing by CHB patient sera deficient in C9

**Fig. 6:**
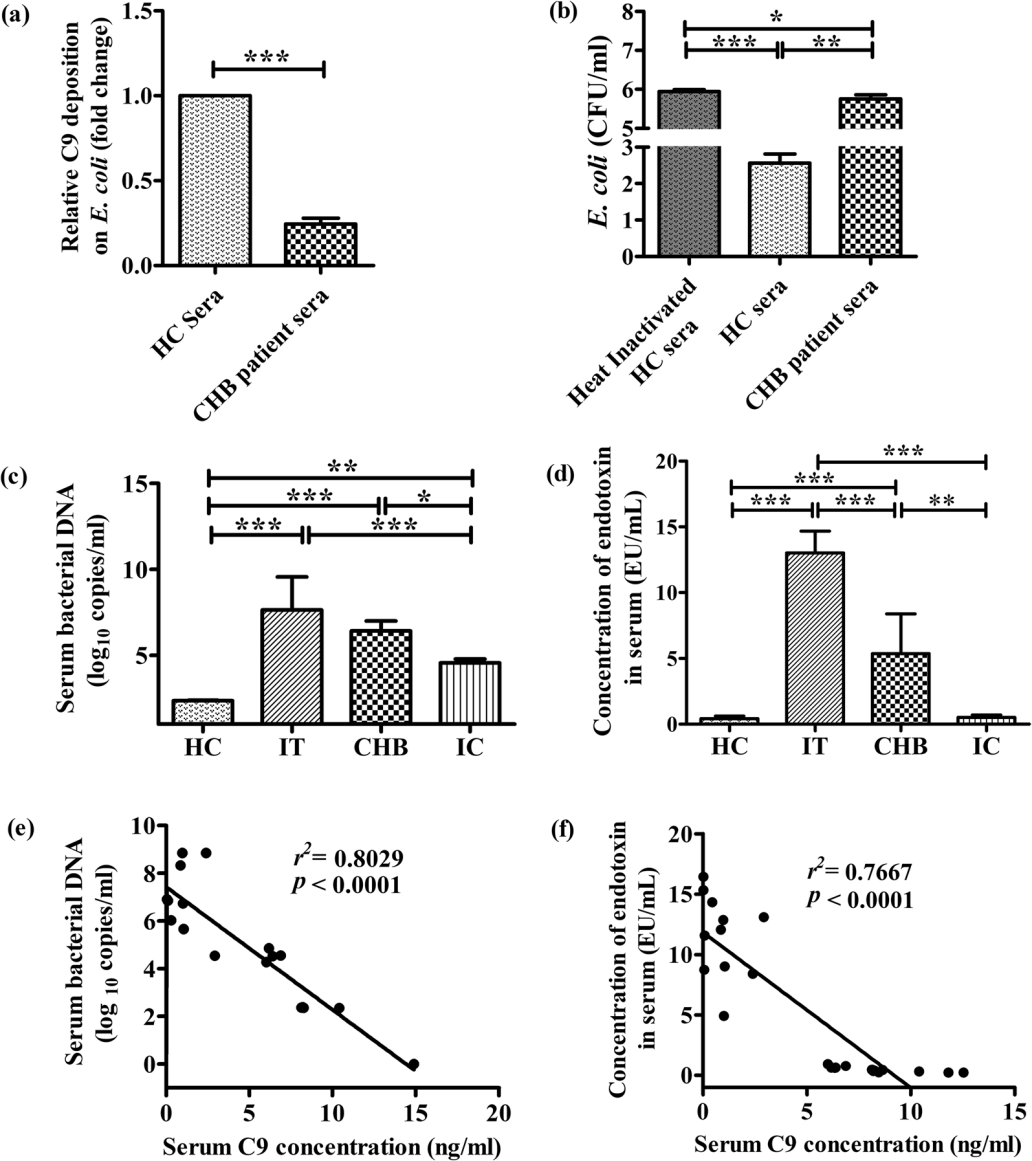
Impairment of MAC mediated bacterial killing by CHB patient sera. **a** C9 deposition on *E. coli* treated with CHB patient sera in comparison to HC sera. **b** CFU count of *E. coli* on agar plates incubated at 37 °C for 16 h after treatment with HC sera, C9 deficient CHB sera or heat inactivated HC sera. Determination of **c** Bacterial-DNA by Real-time PCR and **d** endotoxin concentration by ELISA in the sera of IT, CHB (including both EP- and EN-CHB), IC and HC. Correlation analysis between serum C9 level and **e** bacterial DNA and **f** endotoxin titre in study subjects. Paired *t* test was performed in **a**. One-way ANOVA followed by Tukey’s multiple comparison were used in (b)-(d). *p < 0.05, **p < 0.005, ***p < 0.0001

It has been documented that polymerization of C9 is important for bacterial killing by MAC pores [4]. Having demonstrated the downregulation of C9 in chronically HBV-infected patients, we first studied the level of C9 deposition on the surface of *E. coli* by incubating the bacterial cells with sera of CHB patients or HC and probing with anti-C9 antibody. It was observed that C9 deposit on *E. coli* was ~ 4 fold  lower in case of CHB sera than HC (Fig. 6a). Next, we examined the status of MAC-mediated bacterial killing by enumerating the colonies growing on agar plates following plating of sera treated *E. coli*. Treatment of *E. coli* with heat inactivated HC sera served as negative control. CFU count indicated that HC sera cleared 99% of *E .coli* strain whereas the bacterial clearance was significantly depressed in C9-deficient CHB where only ~ 35% of *E. coli* was cleared (Fig. 6b). Hence the enhanced survival of bacteria could be attributed to the reduced C9 availability and limited MAC formation on their surface.

### Serum bacterial load and endotoxin levels negatively correlate with C9 concentration in HBV-infected patients

To assess the in vivo relevance of our in vitro observations of reduced bactericidal activity of C9 deficient CHB sera, we quantified bacterial-DNA in the sera of chronically HBV-infected patients in IT, EP-/EN-CHB and IC phases and also in HC by Real-time PCR using primers targeting the bacterial 16s  rRNA gene. Serum bacterial-DNA was detected in all study subjects though the concentration varied considerably between the groups. The bacterial-DNA was significantly high in IT and EP-/EN-CHB relative to IC and HC (Fig. 6c). However, IC harboured greater bacterial-DNA than HC. In addition, high serum endotoxin concentration was seen in IT followed by CHB while the titre was much low in IC and HC (Fig. 6d). A clear inverse correlation was perceived between C9 concentration and serum bacterial-DNA load (*r*^*2*^ = 0.8029; *p* < 0.0001) as well as endotoxin level (*r*^*2*^ = 0.7667; *p* < 0.0001) in the study participants (Fig. 6e, f). These findings lead us to postulate that decrease in C9 level was closely associated with high incidence of circulating bacterial-DNA and endotoxin, which could contribute the development of advanced liver diseases in chronically HBV-infected patients.

### Improvement of serum C9 level and decline in viral/bacterial/endotoxin load in Tenofovir-treated CHB patients

**Fig. 7:**
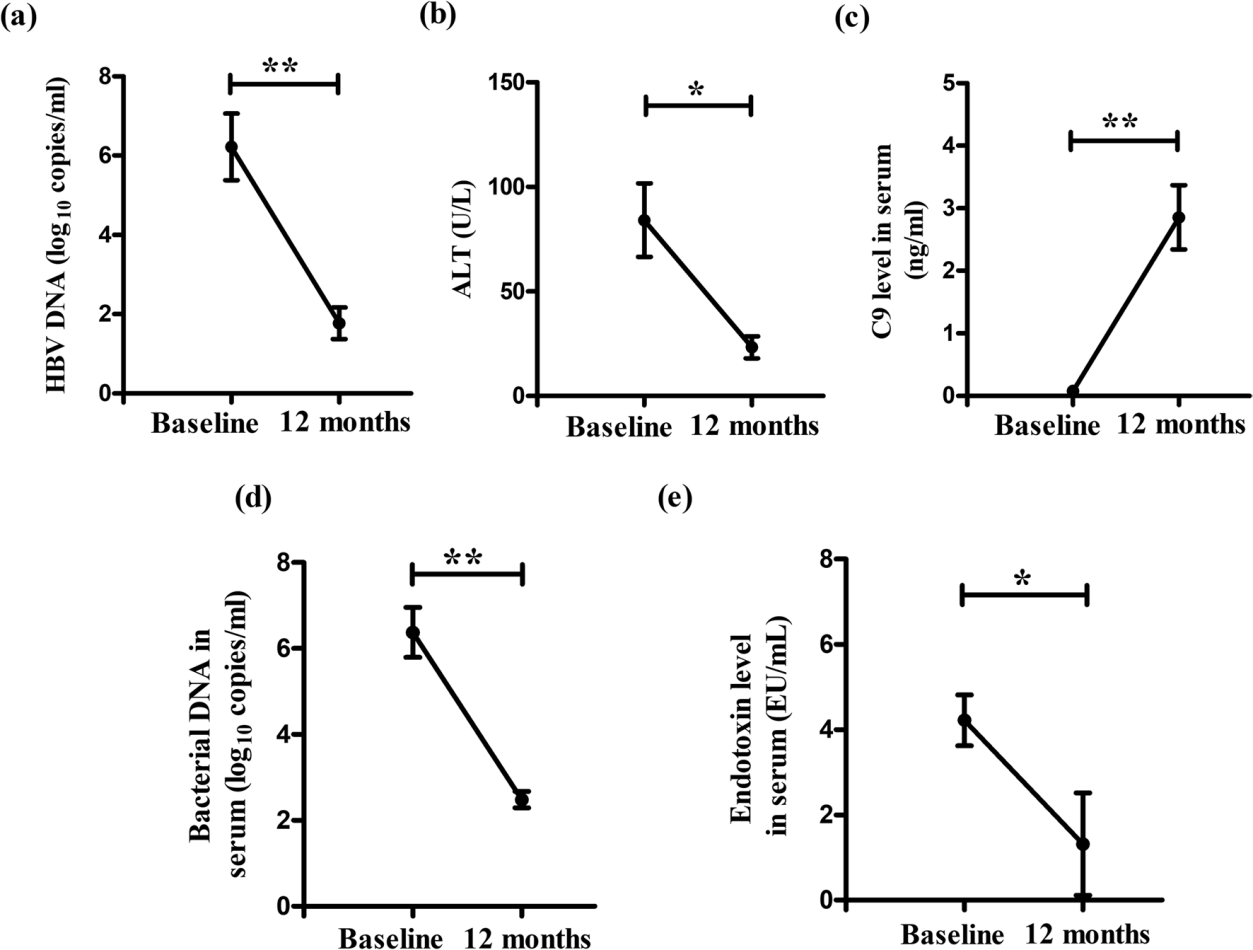
Recovery of serum C9 level and reduction in viral/bacterial/endotoxin load in CHB patients after Tenofovir-therapy. Levels of **a** HBV-DNA, **b** alanine aminotransferase (ALT), **c** C9, **d** bacterial-DNA and **e** endotoxin in sera of CHB patients at baseline and after 12 months of Tenofovir therapy. Wilcoxon matched paired *t* tests were performed. *p < 0.05, **p < 0.005

Tenofovir is recommended as first-line monotherapy for CHB patients [2] and we determined the effect of 1-year of tenofovir treatment on the serum C9 level in 10 CHB patients that included 6 EP-CHB and 4 EN-CHB. We observed that following therapy, all patients achieved < 250 copies/ml of HBV-DNA and normalisation of ALT (Fig. 7a, b) and in parallel there was a marked rise in serum C9 concentration, reduction in bacterial-DNA and endotoxin load as compared to pre-treatment values (Fig. 7c–e).

## Discussion

HBV specifically infects the hepatocytes that are the primary sites of complement synthesis. The chronicity of HBV infection raises the important question as how could HBV subvert the complement-mediated inactivation during CHI. A previous study had reported that HBV by up-regulating the complement regulatory protein CD59 in hepatoma cells restricts the functional MAC assembly and complement-dependent cytotoxicity [18]. However, in the present study, we uncovered a novel mechanism by which HBV interfered with MAC formation by directly targeting the components of MAC. We demonstrated that HBX attenuated the expression of C9, the major component of MAC by impeding the availability of USF-1 that is critical for C9 transcription. HBX-induced upregulation of DNMT activity could be implicated in the aberrant hypermethylation of CpG islands in the USF-1 promoter region thereby silencing USF-1 transcription. The loss of expression of USF-1 and consequently reduction in its transcriptional activity hindered C9 production that in turn impaired the formation and antimicrobial functions of MAC, leading to HBV persistence and high viremia coupled with high incidence of bacterial infection and endotoxin level in chronically HBV-infected patients that may impact the disease pathophysiology. These findings would add to the ongoing efforts of gaining a comprehensive understanding of the HBV disease biology and the mechanisms of HBV evolution and persistence during CHI that will guide the design of therapeutics and vaccines for restoring the usual complement activities and facilitate effective HBV control and disease management.

To gain an insight into the effect of HBV on the expression of different MAC components (C5b/C6/C7/C8/C9), we transfected Huh7 cells with full-length HBV as well as infected HepG2^hNTCP^ cells with HBV particles obtained from HepG2.2.15 cells. A marked reduction in C9 expression was observed in both cases while that of other MAC components remained unaltered. Further, transfection with plasmids expressing individual viral protein depicted that HBX could exert a potent repression of C9 expression. The extent of C9 suppression also increased with increasing amount of HBX expression, suggesting that HBX decreased C9 transcriptional activity in a dose-dependent manner. The change in mRNA level of C9 was also in correspondence with the protein expression. Different studies had suggested that HBX is capable of transactivation and transrepression of both viral and cellular genes through either its direct interaction with several different TFs or may modulate transcriptional activity of the target genes through epigenetic modifications [16]. It was noted that treating HBx-transfected cells with 5-Aza-2’-deoxycytidine but not with TSA could restore C9 expression, suggesting the silencing of C9 was methylation-dependent. We further observed that HBX could repress C9-promoter activation and this prompted our search for CpG islands in C9-promoter with the assumption that HBX-induced hypermethylation events at CpG sites may contribute to reduced promoter activity and consequently failure to transcribe the downstream gene. However the lack of CpG island in C9-promoter led us to explore the alternative possibility that changes in the availability or activity of TFs binding to C9-promoter may be related to the decrease in C9-promoter activity in HBX-transfected cells. We identified by in silico analysis the basic-helix-loop-helix leucine zipper TF, USF-1 as a key regulator of C9 gene expression and a striking reduction in the abundance of endogenous USF-1 was found in HBX-expressing Huh7 cells. Moreover, overexpression of USF-1 was capable of rescuing the repressive effects of HBX on promoter activation and expression of C9. By mutating the E-box motif on C9-promoter and by ChIP assay, we established that USF-1 bound to E-box play a critical role in inducing C9 transcription. We further, elucidated the molecular mechanism by which HBX alters the expression of USF-1. It was noted that treatment with 5-Aza-2’-deoxycytidine could result in modest enhancement of USF-1 transcription in HBX-transfected cells with accompanying increase in C9 expression, implying that HBX-induced DNA hypermethylation is associated with transcriptional repression of USF-1. Park et al*.* [19] had demonstrated that HBX expression could elevate the overall intracellular DNMT activity by inducing DNMT1 and DNMT3A in human liver cell lines and produce aberrant DNA methylation patterns. Consistent with these findings, we also observed high expression of DNMT3A, which functions as de novo methyltransferases in HBX-transfected cells. In addition, localized CpG repeat sequences were detected in the endogenous promoter region of USF-1 gene while bisulfite sequencing revealed extensive methylation of these CpG sites in HBx-transfected Huh7 cells. These and the above-noted results underscore the notion that the major decrease in C9 during CHI was due to downregulation in the expression of the transcription factor USF-1 achieved through HBX-induced aberrant USF-1 promoter methylation. This resulted in a prominently decreased binding of USF-1 to the E-box elements of the C9-promoter thereby diminishing C9 expression.

Together with in vitro experiments, we also studied the levels of serum C9 in chronically HBV-infected patients representing different disease phases. Patients in IT and EP-/EN-CHB phases characterized by high HBV-DNA level were found to exhibit the most pronounced serum C9 deficiency as compared to IC, having low viral load and a strong inverse correlation was seen between C9 concentration and viral load in the study subjects. This suggests a dependence on C9 and MAC for the reduction of the viral titer in chronically HBV-infected patients. In case of Zika virus infection, formation of MAC on viral surface and complement-mediated lysis had been reported to be essential for the reduction of viral RNA [20]. Further, C9 depleted CHB serum also displayed attenuated antimicrobial effect as evident from reduced MAC formation on HBV-transfected Huh7 cells and greater cellular viability along with inefficient killing of bacterial cells following incubation of these cells with CHB sera. However, reconstitution of CHB serum with purified C9 protein led to reversal of these effects. It had been previously documented that HBV-infected patients were co-infected with *E. coli, S. aureus*, *P. aeruginosa* and *K. pneumoniae* and patients with high HBV-DNA had substantially higher rates of concomitant bacterial infections, compared to those with low viral load [10]. In line with this observation, we also noted significant elevation of bacterial-DNA and endotoxin in IT and CHB patients with high viral load and this could be ascribed to the profound deficiency in C9 expression in these patients that is potentiated by enhanced expression of HBX. The bacterial products are recognized by Toll-like and NOD-like receptors on liver cells such as, Kupffer cells and hepatic stellate cells and lamina propria that initiate and maintain inflammatory cascades, which contributed to liver damage [21]. A study examining mRNA expression profiles of liver tissue samples derived from patients with HCC and those with cirrhosis of various etiologies (including HBV and HCV infection) had reported that the downregulation of C9 could be considered as a cancer associated molecular signature in premalignant cirrhotic tissues and may serve as an early diagnostic marker for the onset of HCC [22]. Hence both EP-/EN-CHB and IT with C9 deficiency are at risk for developing HCC while high bacterial infection and endotoxin concentration in these patients could be potential triggers of liver injury. However, 1 year of Tenofovir therapy resulted in substantial decline in HBV-DNA and improvement in C9 levels in CHB patients as well as enhancement in their ability to contain bacterial infection.

## Conclusions

Collectively, we defined a novel mechanism by which HBV inhibits MAC formation and its lytic function in chronically HBV-infected patients, particularly in IT and EP-/EN-CHB phases. Early therapeutic interventions should be considered not only for CHB but also for IT-phase patients to restrict viral replication and expression of HBX and restore the usual complement activities for attaining sustained viral control, decreased vulnerability for bacterial infection and minimize disease pathogenesis.

## Supplementary Information

**Additional file 1.** **Table S1. **List of primers used for amplification, sequencing and cloning of HBV. **Table S2. **List of primers and oligos targeting host genes and promoters. **Table S3. **Clinical, demographic and biochemical data of the study subjects.** Fig S1.** Relative mRNA expression of USF-1 in HBx transfected Huh7 cells treated with or without histone deacetylation inhibitor Trichostatin A (0.1μM).

## Data Availability

The raw data supporting the conclusions of this study will be made available by the authors, without undue reservation, to any qualified researcher.

## Abbreviations

HBV: Hepatitis B Virus
CHI: Chronic HBV infection
MAC: Membrane-attack complex
C9: Complement C9
C5: Complement C5
C6: Complement C6
C7: Complement C7
C8A: Complement C8 alpha chain
C8B: Complement C8 beta chain
ORF: Open reading frame
HBX: Hepatitis B virus X protein
mRNA: Messenger RNA
CHB: Chronic hepatitis B
IT: Immune-tolerant
EP-CHB: HBeAg-positive CHB
IC: Inactive carrier
EN-CHB: HBeAg-negative CHB
USF-1: Upstream transcription factor-1
ATF2: Activating transcription factor 2
FOXO4: Forkhead box O4
STAT1β: Signal transducer and activator of transcription 1- beta
ASO: Antisense oligonucleotides
HCC: Hepatocellular carcinoma
HBsAg: Hepatitis B surface antigen
HBc: Hepatitis B core protein
HBeAg: Hepatitis B e antigen
HBV-P: Hepatitis B polymerase
HIV: Human immunodeficiency virus
HC: Healthy individuals
ChIP: Chromatin immunoprecipitation
DAPI: 4′,6-Diamidino-2-phenylindole
EIA: Enzyme immunoassay
HRP: Horseradish peroxidase
rRNA: Ribosomal RNA
PCR: Polymerase chain reaction
ELISA: Enzyme-linked immunoassay
DNMT: DNA methyltransferase
CFU: Colony forming unit
ALT: Alanine aminotransferase
TF: Transcription factor
E-box: Enhancer box
HRP: Horseradish peroxidase
DAPI: 4′,6-Diamidino-2-phenylindole
SD: Standard deviation
ANOVA: Analysis of variance
