# Supplementary material for: Hepatitis B virus suppresses complement C9 synthesis by limiting the availability of transcription factor USF-1 and inhibits formation of membrane attack complex: implications in disease pathogenesis
Source: J Biomed Sci. 2022 Nov 14;29:97. doi: 10.1186/s12929-022-00876-1 (PMC9664717; doi:10.1186/s12929-022-00876-1)
Supplement: Supplementary file 1 — Additional file 1. Table S1. List of primers used for amplification, sequencing and cloning of HBV. Table S2. List of primers and oligos targeting host genes and promoters. Table S3. Clinical, demographic and biochemical data of the study subjects. Fig S1. Relative mRNA expression of USF-1 in HBx transfected Huh7 cells treated with or without histone deacetylation inhibitor Trichostatin A (0.1μM). [file 12929_2022_876_MOESM1_ESM.doc]

**Supplementary Information**

**Hepatitis B virus suppresses complement C9 synthesis by limiting the availability of transcription factor USF-1 and inhibits formation of membrane attack complex: implications in disease pathogenesis**

Ayana Baidya^1^, Mousumi Khatun^1^, Rajiv Kumar Mondal^1^, Suchandrima Ghosh^1^, Bidhan Chandra Chakraborty^2^, Shreya Mallik^1^, SK Mahiuddin Ahammed^3^, Abhijit Chowdhury^3^, Soma Banerjee^1^,

Simanti Datta^1^*

^1^Centre for Liver Research, School of Digestive and Liver Diseases, Institute of Post Graduate Medical Education and Research (I.P.G.M.E. & R.), Kolkata, India

^2^Multidisciplinary Research Unit, Institute of Post Graduate Medical Education and Research (I.P.G.M.E. & R.), Kolkata, India

^3^Department of Hepatology, School of Digestive and Liver Diseases, Institute of Post Graduate Medical Education and Research (I.P.G.M.E. & R.), Kolkata, India

***Supplementary Table S1: List of primers used for amplification, sequencing and cloning of HBV***

| **Primer Name** | **Primer Sequences** |
| --- | --- |
| HBVP1^a,#^ (Sense) | 5′-CCGGAAAGCTTGAGCTCTTCTTTTTCACCTCTGCCTAATCA -3′ |
| HBVP2^a,#^ (Antisense) | 5′-CCGGAAAGCTTGAGCTCTTCAAAAAGTTGCATGGTGCTGG -3′ |
| HBp_F^b^ (Sense) | 5’ TACTGGTACCGTGTGGATTCGCACTCC 3’ |
| HBp_R^b^ (Antisense) | 5’ TGTAAAGCTTGAGAGTCCAAGAGTCCTC 3’ |
| HBsHindIII_F^c^ (Sense) | 5'-TACTAAGCTTATGGGGCAGAATC-3' |
| HBsPstI_R^c^ (Antisense) | 5'-TACACTGCAGAATGTATACCCAAAG-3' |
| F10^d^ (Sense) | 5' -GACCACCAAATGCCCCTATC -3’ |
| F3^d^ (Sense) | 5’-CGCCTCATTTTGTGGGTCAC-3’ |
| F4^d^ (Sense) | 5’-CTCAGGCCATGCAGTGGAA- 3’ |
| CF^d^ (Sense) | 5’-ACTGTTCAAGCCTCCAAGCT-3’ |
| R2^d^ (Antisense) | 5’-AAATTACCACCCACCCAGG-3’ |
| R3^d^ (Antisense) | 5’-AACTGGAGCCACCAGCAG- 3’ |
| R5^d^ (Antisense) | 5'-AAAGCCCAAAAGACCCACAAT-3' |
| R9^d^ (Antisense) | 5´-TAGGAGTTCCGCAGTATGGA- 3´ |
| R10^d^ (Antisense) | 5’-CAGCCTCCTAGTACAAAGAC-3’ |
| F7^d^ (Sense) | 5’-TGTGCACTTCGCTTCACCTC-3’ |

^a^Primers used for full genome amplification of HBV, ^#^Günther S, et al. J Virol 1995;69:5437-44. ^b^Primers used for complete HBV-polymerase amplification for cloning. ^c^Primers used for complete PreS1/PreS2/S-ORF amplification for cloning. ^d^Primers used for sequencing HBV genome.

***Supplementary Table S2: List of primers and oligos targeting host genes and promoters***

| **Primer Name** | **Primer Sequences** | **Purpose** |
| --- | --- | --- |
| C5F (sense) | 5’ TCACTGGAGACTTGGTTTGG 3’ | Measurement of C5 mRNA expression by Real-time PCR |
| C5R (antisense) | 5’ ACTCCTTTCGTCTGCTAATGG 3’ |
| C6F (sense) | 5’ GACTGTAGCCATCATTCAG 3’ | Measurement of C6 mRNA expression by Real-time PCR |
| C6R (antisense) | 5’ CTTGGCAGGAACCAATATG 3’ |
| C7F (sense) | 5’CTACGAATGTGGACCTTC 3’ | Measurement of C7 mRNA expression by Real-time PCR |
| C7R (antisense) | 5’GCTGTCCCTACCAGTAAG 3’ |
| C8AF (sense) | 5’ GTAAGGAGACAGGTCGCTG 3’ | Measurement of C8A mRNA expression by Real-time PCR |
| C8AR (antisense) | 5’ CTGGAATTGGTTCATACTGG 3’ |
| C8BF (sense) | 5’ GTGCACAGACAGGAAGGTG 3’ | Measurement of C8B mRNA expression by Real-time PCR |
| C8BR (antisense) | 5’ GGTCCATTTCATGCTGAC 3’ |
| C9F (sense) | 5' CATGAAGCGGAAAGGTGTTG 3’ | Measurement of C9 mRNA expression by Real-time PCR |
| C9R (antisense) | 5’ CAGCTCCAACAGAGATTTCAG 3’ |
| C9-prom_F | 5’- TACTGGTACCTTCAAAATGTACCACTCGGCCTATAAG -3’ | Cloning of C9 promoter |
| C9-prom_R | 5’- TGTAAAGCTTAAATGCAGATTGCAACTGCAAAGC – 3’ |
| C9-prom_ChIP_F | 5’- GTACCACTCGGCCTATAAGG -3’ | Amplification of C9 promoter after ChIP assay |
| C9-prom_ChIP_R | 5’-CTGCACTTAGTGGAAAGGATGG-3’ |
| C9_del_56-61_F | 5'-GGATTGTAAATGCATTTGCTGAAATAACCTACCTAGCCC-3' | Generation of C9-Prom_mt construct by SDM |
| C9_del_56-61_R | 5'-GGGCTAGGTAGGTTATTTCAGCAAATGCATTTACAATCC-3' |
| USF-1 F | 5’-GAGTACAGCTGCTGTTGTTAC-3’ | Measurement of USF-1 mRNA expression by Real-time PCR |
| USF-1 R | 5’-TGCAGTACTTCTTGTGGTGAC-3’ |
| BS_USF1P_F(NEW) | 5’GTTGGTTTTTTATTTTATATTTTGTTTTG 3’ | Bisulfite sequencing of USF-1 promoter |
| BS_USF1P_R2(NEW) | 5’ CCAATAAAARCCCTTRCTAAATCTAAACAC 3’ |
| USF1_ASO | 5’-CAGCTGTTTTCTGCTGCCCCTTCAT-3’ | Antisense oligonucleotide against exon1 of USF-1 used for transfection |
| ASO-Ctrl^#^ | 5’- TAGTGCGGACCTACCCACGA-3’ | Scrambled oligo  control used for transfection |

^#^ Billioud G, et al. J Hepatol. 2016;64(4):781-9.

**Supplementary Table S3:** Clinical, demographic and biochemical data of the study subjects

|  | **HC (n=20)** | **IT (n=7)** | **EP-CHB (n=16)** | **IC (n=20)** | **EN-CHB (n=12)** |
| --- | --- | --- | --- | --- | --- |
| **Age (Years),**  **Median (Range)** | 31 (25-43) | 12 (7-26) | 30 (18-48) | 35 (23-50) | 23 (20-60) |
| **Sex (Male:Female)** | 13:7 | 5:2 | 12:4 | 16:4 | 9:3 |
| **ALT (IU/L),**  **Median (Range)** | 22 (16-30) | 32 (19-37) | 74 (54-110) | 25 (17-38) | 62 (42-210) |
| **AST (IU/L),**  **Median (Range)** | 21 (17-33) | 31 (11-39) | 56 (42-174) | 25 (22-40) | 61 (40-170) |
| **HBeAg status** | - | Positive | Positive | Negative | Negative |
| **HBV DNA**  **(copies/mL)**  **Median (Range)** | - | 4.6X10^8^  (2.7X10^7-^2.3X10^9^) | 1.8X10^6^  (1.4X10^5^-3.5X10^7^) | 250  (250-4.8X10^3^) | 2.2X10^5^  (1.1X10^4-^9.2X10^6^) |

IT, Immunotolerant; EP-CHB, HBeAg-positive chronic hepatitis B; IC, Inactive carriers; EN-CHB, HBeAg-negative chronic hepatitis B; HC, Healthy controls; ALT, alanine aminotransferase; AST, aspartate aminotransferase; IU, international unit.

***Antibodies***

| **Antibody** | **Catalogue No.** |
| --- | --- |
| Anti-C9 antibody | sc-390000 |
| Anti-C5b-9 antibody | sc-58935 |
| Anti-USF-1 antibody | sc-390027 |
| Anti-α-tubulin antibody | sc-398103 |
| Goat anti-Mouse IgG secondary antibody, HRP | 32430 |
| Goat anti-Mouse IgG secondary antibody, Alexa fluor 488 | A11017 |

**Supplementary Figure:**


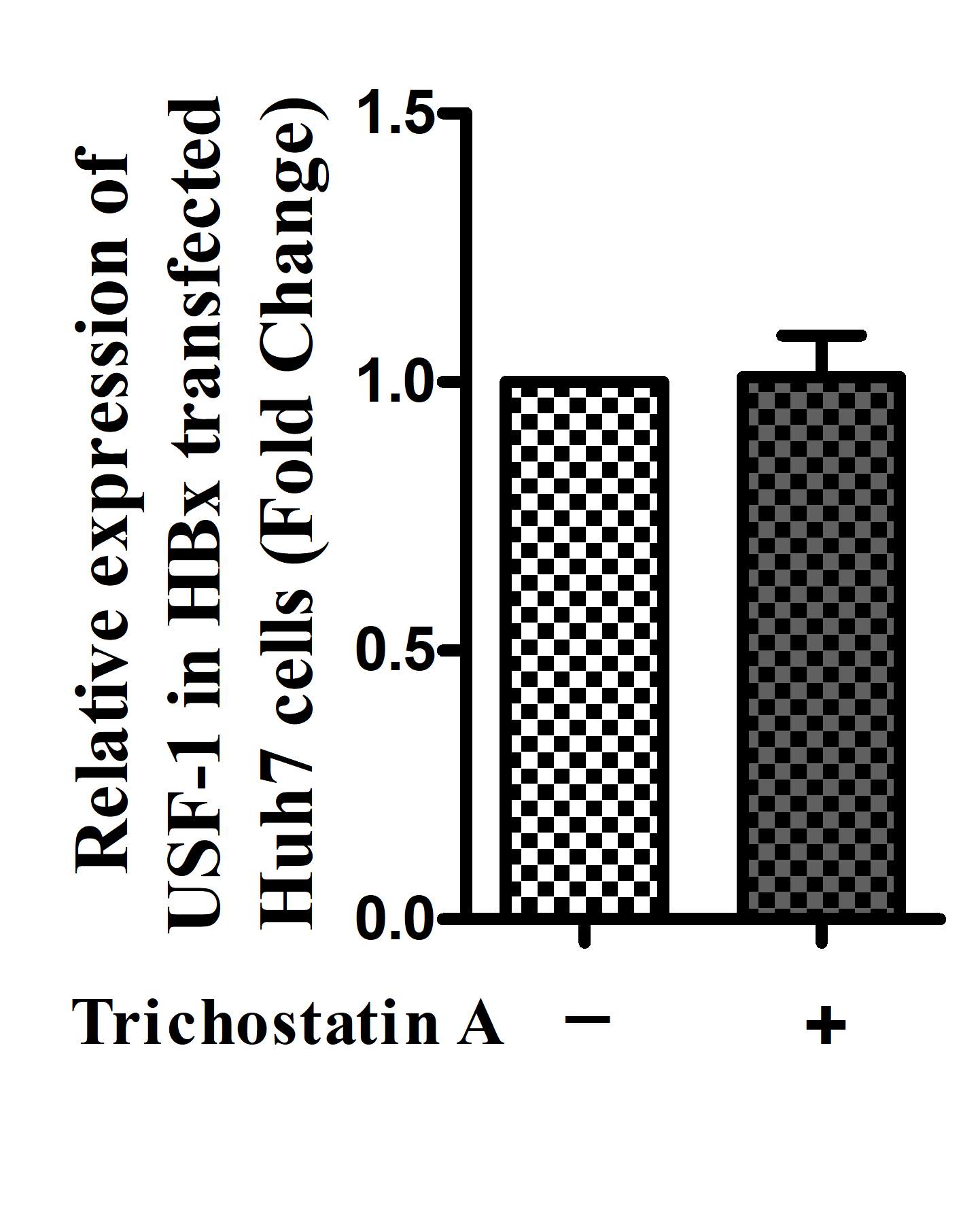


**Fig S1:** Relative mRNA expression of USF-1 in HBx transfected Huh7 cells treated with or without histone deacetylation inhibitor Trichostatin A (0.1μM).
